# A Sigfox Energy Consumption Model

**DOI:** 10.3390/s19030681

**Published:** 2019-02-07

**Authors:** Carles Gomez, Juan Carlos Veras, Rafael Vidal, Lluís Casals, Josep Paradells

**Affiliations:** Department of Network Engineering, Universitat Politècnica de Catalunya/Fundació i2Cat, C/Esteve Terradas, 7, 08860 Castelldefels, Spain; vjcharlie5@gmail.com (J.C.V.); rafael.vidal@entel.upc.edu (R.V.); lluis.casals@entel.upc.edu (L.C.); josep.paradells@entel.upc.edu (J.P.)

**Keywords:** Sigfox, energy, modeling, performance evaluation, Internet of Things, IoT, smart cities, LPWAN

## Abstract

Sigfox has become one of the main Low-Power Wide Area Network (LPWAN) technologies, as it has attracted the attention of the industry, academy and standards development organizations in recent years. Sigfox devices, such as sensors or actuators, are expected to run on limited energy sources; therefore, it is crucial to investigate the energy consumption of Sigfox. However, the literature has only focused on this topic to a very limited extent. This paper presents an analytical model that characterizes device current consumption, device lifetime and energy cost of data delivery with Sigfox. In order to capture a realistic behavior, the model has been derived from measurements carried out on a real Sigfox hardware module. The model allows quantifying the impact of relevant Sigfox parameters and mechanisms, as well as frame losses, on Sigfox device energy performance. Among others, evaluation results show that the considered Sigfox device, powered by a 2400 mAh battery, can achieve a theoretical lifetime of 1.5 or 2.5 years while sending one message every 10 min at 100 bit/s or 600 bit/s, respectively, and an asymptotic lifetime of 14.6 years as the message transmission rate decreases.

## 1. Introduction

Low-Power Wide Area Networks (LPWANs) have recently emerged as a category of wireless technologies suitable for enabling Internet of Things (IoT) applications in a diversity of domains [1]. LPWAN technologies have been designed to support low energy consumption, since IoT devices (e.g., sensors and actuators) often rely on a limited energy source, such as a battery. However, in contrast with the rather short range of many established IoT technologies [2], LPWAN technologies provide an extended link range of up to several kilometers. Furthermore, a single radio gateway can offer network connectivity to hundreds of thousands of IoT devices. In consequence, LPWAN requires a low amount of infrastructure to be deployed and maintained, which has fueled its momentum in recent years.

One of the most popular LPWAN technologies is called Sigfox [3]. This technology was developed in 2009 by the Sigfox company (Labège, France). As of the writing, Sigfox offers network coverage allowing bidirectional communication for IoT devices in more than 50 countries, covering a population of 1 billion inhabitants. Currently, the IETF LPWAN working group is developing functionality to support IPv6 over Sigfox, thus enabling Internet connectivity for Sigfox devices [4,5].

Many academic studies focus on evaluating the performance of Sigfox [6,7,8,9,10,11,12,13,14,15]. Since Sigfox devices, such as sensors or actuators, are expected to run on constrained energy sources, investigating the energy consumption of Sigfox is crucial. However, as of the writing, this topic has received limited attention, and existing models in the literature [9,10,11,12,13,14,15] are too simple (see Section 2). 

This paper analytically models device current consumption, device lifetime and energy efficiency of data delivery with Sigfox. In order to capture a realistic behavior, our model is based on measurements conducted on a real hardware platform. The model presented allows to determine the influence of crucial Sigfox parameters and mechanisms, such as the uplink physical layer data rate, payload size, unidirectional or bidirectional communication, and message losses, on Sigfox energy performance. Our evaluation results illustrate the energy performance and trade-offs of Sigfox. Among others, we have found that a Sigfox device running on a 2400 mAh battery can achieve a lifetime of 1.5 years (at 100 bit/s) or 2.5 years (at 600 bit/s) while transmitting one uplink message every 10 min, and an asymptotic theoretical lifetime of 14.6 years as the message sending rate decreases. 

The remainder of the paper is organized as follows: Section 2 reviews related work. Section 3 overviews Sigfox, describing its network architecture and its main communication mechanisms. Section 4 models Sigfox end-device current consumption, end-device lifetime, and energy cost of data delivery. Section 5 evaluates and discusses the results obtained by using the model presented, assesssing also the sensitivity of the model to relevant parameters, and the use of energy harvesting sources. Finally, Section 6 concludes the paper.

## 2. Related Work

Many published works focus on Sigfox performance evaluation. Relevant performance parameters considered by researchers include coverage, capacity and energy consumption [6,7,8]. While the latter is crucial, considering that many Sigfox devices are expected to run on constrained energy sources, it has not been accurately or comprehensively addressed. Next, a review of the literature on Sigfox energy consumption is provided.

As of the writing, a few analytical models of Sigfox current consumption, device lifetime or energy cost of data delivery have been published [9,10,11,12,13,14,15]. These models are too simple, since they do not capture all the current consumption states related with data transmission or reception on a real Sigfox device (see Table 1). Furthermore, they do not adequately consider (or do not consider at all) frame losses. Other relevant parameters ignored by some of these models include the uplink bit rate and the uplink frame payload size. A brief review of the main features, results and limitations of these models is given next. 

Morin et al. present a device lifetime comparison for a wide range of wireless IoT technologies [9]. Assuming two AAA batteries (of 1250 mAh each), values up to 15 and 25 years are derived for Sigfox when only 10 bytes per day are sent at 100 bit/s and 1 kbit/s, respectively. The model provided only considers uplink communications, and only two power consumption states (i.e., transmission and sleep) are considered. Furthermore, how the current consumption values for these states are obtained is not clear. Finally, impact of errors on device lifetime is modeled by assuming frame retransmission based on lack of confirmation, while Sigfox uses a completely different approach (see Section 3.2). A similar comparison study focusing on LPWAN technologies is presented in [10]. Assuming a 5 Wh battery, lifetime values up to around 100 years are estimated for a device sending one byte of data per day. The analytical model in that study does not capture all power consumption states, and their characterization is not based on real hardware measurements. In addition, authors only consider uplink communication, and fixed settings for the frame loss rate and the bit rate are used. How frame losses are handled in the model is not specified. These two papers report a long device lifetime since they assume an ideal sleep current, one order of magnitude below the one measured in our paper (see Table 1 and Table 2).

Hernández et al. provide an analytical model that considers uplink and downlink communications, with a characterization of power consumption states based on measurements carried out on a real platform [11]. Using a 1 Ah battery, a maximum device lifetime of 9 years and 1.2 years is estimated for uplink and downlink communication, with 2 h between consecutive transmissions. The model defines less power consumption states than the one presented in our paper, and state durations appear to be rounded to hundreds of milliseconds. The model does not consider the impact of frame losses, misses the fact that the downlink window duration is random (see Section 3), uses a fixed bit rate (of 100 bit/s) and does not study the impact of the payload size.

The rest of papers considered in this literature review provide an analytical energy consumption model, but they do not use it to estimate the device lifetime. Ogawa et al. present an analytical model for confirmed uplink communications, with the goal of estimating the energy cost of data delivery per year [12]. Only transmission and reception power consumption states are considered and their current consumption values are obtained from the datasheet of a module. The result is therefore a very simplistic model. In addition, the impact of the bit rate, errors or the frame payload size are not taken into account. Ruckebusch et al. follow a similar approach, with the same limitations, in order to compare the behavior of different LPWAN technologies for over-the-air software updates [13]. Using this model in a confirmed uplink communication, the energy cost of delivering a 12-byte frame payload is estimated to be around 0.4 J. In another work, the objective of the analytical model presented is comparing the power consumption of GPS and Sigfox-based localization [14]. Power consumption states are identified empirically. Using the model for an uplink communication at a hypothetic 2-second transmission interval, the energy cost of delivering a frame with 1-byte and 12-byte payloads is estimated as 1.05 and 1.47 Joules, respectively. However, this model is limited to uplink communication, and it does not take in consideration errors or the impact of the uplink bit rate. Finally, Martínez et al. present an analytical model for estimating the current consumption of a device, based on empirical data [15]. However, crucial information, including the device model, the frame payload, the uplink bit rate, the states in the model and their associated duration and current consumption values, is missing. In addition, the analytical model considers neither downlink communication nor the impact of frame losses. 

Based on our literature review, we conclude that our paper is the first one that provides a detailed and comprehensive analytical model of Sigfox device current consumption, device lifetime and energy cost of data delivery, considering real Sigfox device hardware behavior, the different communication settings and mechanisms (uplink/downlink communication, payload size and uplink bit rate), and the impact of frame losses. 

## 3. Sigfox Overview

This section provides an overview of Sigfox. The section is organized in two parts. The first one describes the Sigfox network architecture. The second one focuses on the Sigfox radio interface, covering physical communication characteristics, as well as protocol features. 

### 3.1. Network Architecture

**Figure 1:**
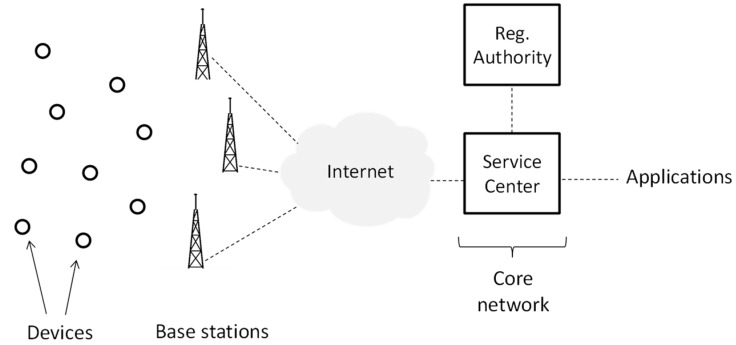
Sigfox network architecture [3].

The Sigfox network architecture comprises devices, base stations and a core network (Figure 1). Devices (e.g., sensors or actuators) are provided with wireless connectivity via neighboring base stations. A device is not bound to a particular base station. Therefore, association signaling is not needed. The base stations are connected through the public Internet with a single cloud-based core network. This approach avoids handover procedures to support device mobility. The core network is composed of the Service Center and the Registration Authority. The Service Center controls and manages the base stations and the devices. The Registration Authority is responsible for authorizing the network access of devices. Applications may interact with the data collected by devices, and with devices themselves, via a web interface and a number of Application Program Interfaces (APIs).

### 3.2. Sigfox Radio Interface

Sigfox supports unidirectional and bidirectional communication over unlicensed spectrum. In Europe, the bands 868.00 MHz–868.60 MHz and 869.40 MHz to 869.65 MHz are used for uplink and downlink transmission, respectively. In the USA, the 902 MHz band is used. In order to achieve a long link range, while limiting the transmit power, Sigfox uses Ultra Narrow Band (UNB) radio transmission for both uplink and downlink. The bandwidth of an uplink channel depends on the region (e.g., it is 100 Hz in Europe and 600 Hz in the United States), while the downlink channel bandwidth is 1.5 kHz. The maximum uplink transmit power is 25 mW in Europe (158 mW in the USA), whereas the maximum downlink transmit power is 500 mW in Europe (4 W in the USA). The modulations used for the uplink and the downlink are Differential Binary Phase-Shift Keying (DBPSK) and Gaussian Frequency-Shift Keying (GFSK), respectively. DBPSK is more bandwidth-efficient than GFSK, which favors an increased uplink range (compensating for the lower permitted transmit power in the uplink band). In addition, DBPSK yields good protection against interference (e.g., jamming), as received power then concentrates in a very narrow bandwidth and reaches a high received power level. The uplink physical layer bit rate is 100 bit/s (in Europe) or 600 bit/s (in the United States) whereas the downlink physical layer bit rate is 600 bit/s worldwide.

Sigfox uses license-free spectrum, which is subject to spectrum access regulations. For example, in Europe, the bands used by Sigfox for uplink and downlink transmission are subject to a duty cycle limitation of 1% and 10%, respectively [6]. In order to comply with spectrum usage regulations, the system typically allows up to 140 uplink messages and four downlink messages per day. These message rate constraints may be relaxed depending on the specific regulatory domain of operation and on system conditions [3].

**Figure 2:**
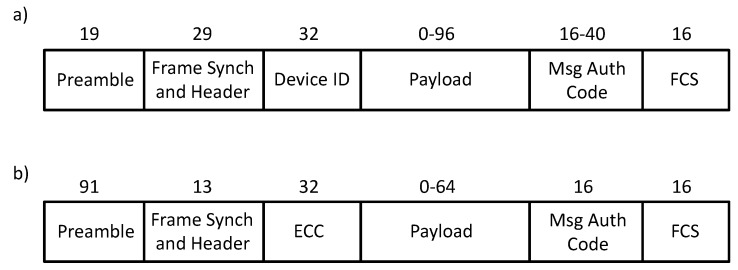
Sigfox frame formats: (**a**) uplink and (**b**) downlink. All frame field sizes are expressed in bits. The third frame header field starting from the left is the Device Identifier (Device ID) or the Error Correcting Code (ECC), for uplink and downlink frame formats, respectively. The two rightmost frame fields are a Message Authentication Code (Msg Auth Code) and a Frame Check Sequence (FCS).

Sigfox defines the physical frame formats for uplink and downlink message transmission (see Figure 2). The minimum uplink and downlink frame sizes are 14 bytes and 21 bytes, respectively. The maximum frame size is 29 bytes for both uplink and downlink.

Communication is asynchronous and device-initiated, which allows the device to stay in sleep state by default and minimize its energy consumption. An uplink message transmission may be received by several base stations (on average, by three base stations [19]), enabling cooperative reception and spatial diversity. This approach naturally supports device mobility.

**Figure 3:**
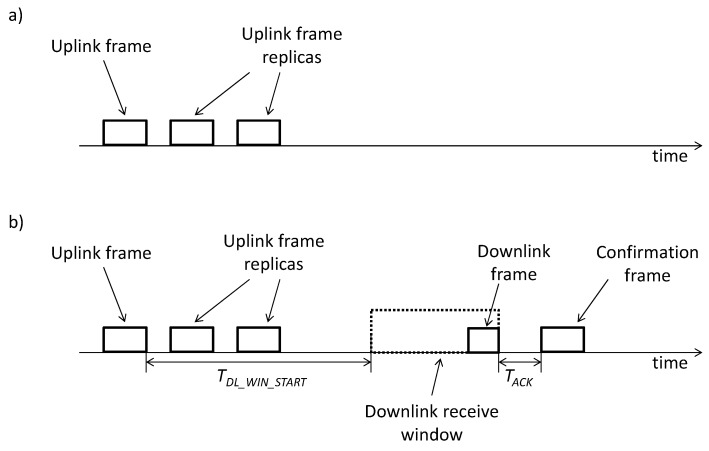
(**a**) Unidirectional and (**b**) bidirectional transactions in Sigfox.

Sigfox defines two types of message exchanges: unidirectional and bidirectional transactions (Figure 3). In the first one, the device transmits an uplink frame via a randomly selected frequency channel, and then transmits two exact replicas of that frame, by using other random frequency channels at different time intervals. This feature provides frequency and time diversity, which contribute to communication robustness in the presence of issues such as multipath fading, interference, etc. Note that, in unidirectional transactions, there is no response to uplink frame transmission. Therefore, unidirectional transactions are unconfirmed. In bidirectional transactions, an uplink message is first transmitted by the device by using the same procedure as in unidirectional transactions (i.e., a first uplink frame is followed by two replicas in different frequency channels). After a time, denoted *T_DL_WIN_START_*, since the end of the first uplink frame transmission, the device initiates a receive window, of maximum duration denoted *T_DL_WIN_MAX_*, intended to enable reception of a downlink frame sent by a base station. The downlink frame may carry actual application data for the device and, at the same time, it may also serve as an acknowledgment for the uplink frame. After reception of the downlink message, an uplink confirmation is sent by the device after *T_ACK_* time. Note that, in contrast with other technologies, retransmissions due to absence of feedback from the other endpoint of a link do not exist in Sigfox.

## 4. Modeling Sigfox Device Current Consumption

This section presents models of crucial energy performance parameters of Sigfox, such as device current consumption, device lifetime (for battery-operated devices), and energy efficiency of data delivery. A device periodically transmits an uplink data message (e.g., a message that carries a sensor reading) is assumed. The models consider the impact of frame losses. The section is divided in two subsections, which offer the aforementioned models for unidirectional and bidirectional transactions, respectively. All Sigfox physical layer bit rates are considered. 

### 4.1. Unidirectional Transactions

**Figure 4:**
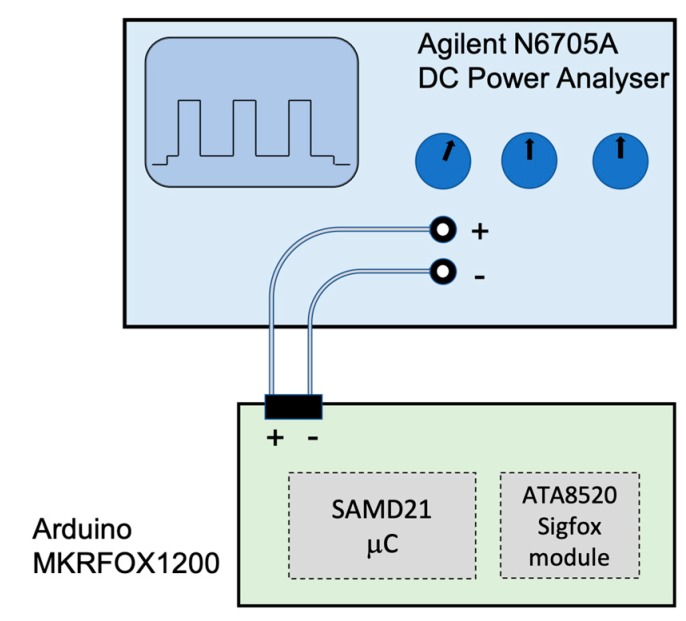
Experimental setup for the current consumption characterization of the MKRFOX1200 Sigfox module (on the left) using an Agilent N6705A power analyzer.

We first model the average current consumption of a device communicating by means of undirectional exchanges, denoted *I_avg_uni_*. To this end, a profile of the different states involved in a unidirectional transaction is created, considering the duration and current consumption of each state. In order to realistically model the device current consumption characteristics, and without loss of generality, the model is developed based on measurements from a real Sigfox device. Figure 4 shows the measurement setup, which includes an N6705A power analyzer (Agilent, Santa Clara, CA, USA) and an MKRFOX1200 Sigfox device (Arduino, Somerville, MA, USA) [20]. The voltage supplied by the power analyzer is 3 V. The transmit power of the device is 14.5 dBm. The uplink bit rate supported by the device is 100 bit/s. The device is located in an indoor scenario, and makes use of the network coverage provided by Sigfox in the Barcelona area. A 16-bit Msg Auth Code field is used in uplink frames.

The Sigfox device is assumed to periodically perform a transaction, therefore its current consumption behavior is modeled over one period. In this subsection, unidirectional transactions are assumed. Therefore, each period comprises the operations carried out by the device to perform the transmission of one data message, including its replicas, whereas the device is in sleep mode during the remaining time in the period. Note that, in unidirectional transactions, device current consumption is independent of packet losses. Time and current consumption measurement results are obtained from several individual measurements within a transaction period. Negligible differences were found within each set of individual measurements. 

**Figure 5:**
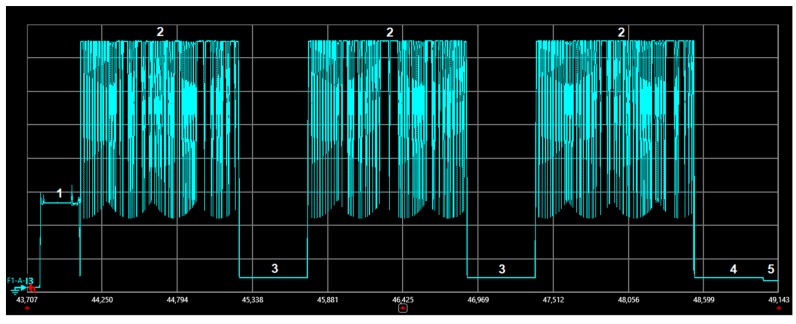
Current consumption profile of a MKRFOX1200 device performing a unidirectional transaction. The data message transmitted has a payload size of 1 byte.

Figure 5 depicts the current consumption profile of a unidirectional transaction performed by the MKRFOX1200 device. Table 2 indicates the different states involved in a unidirectional transaction, as well as the variables used to denote the corresponding duration and current consumption of each state. The state duration and current values shown in Table 2 correspond to the average of 10 individual measurements in each case, with a maximum observed deviation from the average value below 5%. The initial state of the device is the sleep state, where the device current consumption is 2–3 orders of magnitude below that of the rest of states. Note that this measured sleep current is greater than that obtained from transceiver datasheets (e.g., [9,10]). A similar phenomenon was observed for LoRa/LoRaWAN devices [21]. A reason is that, on devices such as development kits, measured sleep current is the sum of the sleep currents from all circuits including the transceiver, microcontroller, plus the sum of the leakage currents of decoupling and filtering capacitors, as well as resistors. In order to initiate the procedure for carrying out the message transmission, the device first wakes up (state 1). Next, the device performs the transmission of the first uplink frame transmission (state 2). After that, it waits for an interval (state 3) that ends with the start of the transmission of the first uplink frame replica (state 2). Similarly, another wait time (state 3) and a second replica transmission (state 2) follow. Finally, the device executes a cool down sequence (state 4) before returning to the sleep mode (state 5). 

Let *T_Period_* denote the time between two consecutive periodic transactions initiated by the device. Let *T_i_* and *I_i_* represent the duration and current consumption of state *i* in Table 1, respectively. Based on the introduced variables, the average current consumption for a device performing unidirectional transactions, *I_avg_uni_*, can be obtained as per Equation (1):

(1) $$I_{avg\_uni}=\frac{1}{T_{Period}}\sum_{i=1}^{N_{states\_uni}}n_{i}\cdot T_{i}\cdot I_{i}$$

where *n_i_* indicates the number of times state *i* is present in the uplink data frame transmission procedure (note that *n*_2_ = 3, *n*_3_ = 2, and *n_i_* = 1 otherwise) and *N_states_uni_* is 5. Note that *T_sleep_* can be computed as:

(2) $$T_{sleep\_uni}=T_{Period}-T_{act\_uni}$$

where *T_act_uni_* denotes the sum of the durations of all states related with transmission activities, i.e., a wake-up state (of duration *T_wu_*), 3 transmission states (each one of duration *T_tx_*), 2 wait next transmission states (of duration *T_wntx_*) and the cool down state (of duration *T_cd_*):

(3) $$T_{act\_uni}=T_{wu}+3\cdot T_{tx}+2\cdot T_{wntx}+T_{cd}$$

The transmission time, *T_tx_*, is variable and depends on the total uplink frame size (*l_frame_UL_*), and on the uplink bit rate in use (*BR_UL_*):

(4) $$T_{tx}=\frac{l_{frame\_UL}}{BR_{UL}}$$

As *I_avg_uni_* can be computed by using Equations (1)–(4), the theoretical lifetime of a battery-operated device that performs unidirectional transactions, denoted *T_lifetime_uni_*, can be determined as shown next, by taking into account the battery capacity, *C_battery_* (expressed in mA·h), and by approximating a realistic battery behavior by considering the battery self-discharge current, *I_self_dis_*:

(5) $$T_{lifetime\_uni}=\frac{C_{battery}}{I_{avg\_uni}+I_{self\_dis}}\;$$

where *I_self_dis_* is assumed as a constant value over time. 

The third performance parameter modeled is the energy cost of data delivery, *EC_delivery_uni_*, which indicates the energy consumed by the device per each successfully delivered bit of data payload in unidirectional transactions. *EC_delivery_uni_* can be obtained as shown next:

(6) $$EC_{delivery\_uni}=\frac{I_{avg\_uni}\cdot V\cdot T_{Period}}{E\left[l_{delivery}\right]}$$

where *V* denotes the battery voltage and *E*[*l_delivery_*] indicates the expected amount of data delivered by the device. In the previous equation, the numerator indicates the energy consumed by the device during *T_Period_*. 

In unidirectional transactions, the current consumed by the device and its lifetime are independent of uplink frame delivery success. However, *E*[*l_delivery_*] depends on the Frame Loss Rate (FLR), and therefore frame losses have an impact on *EC_delivery_uni_*. Let *l_Payload_* be the frame payload size, and let *FLR_UL_* denote the FLR at the base station for a single uplink frame transmission. The uplink data message will be correctly delivered if at least one of the corresponding three uplink frame transmissions is successfully received. Then, the expected amount of data delivered by the device per transaction is determined as:

(7) $$E\left[l_{delivery}\right]=l_{Payload}\cdot \left(1-FLR_{UL}{}^{3}\right)$$

### 4.2. Bidirectional Transactions 

**Figure 6:**
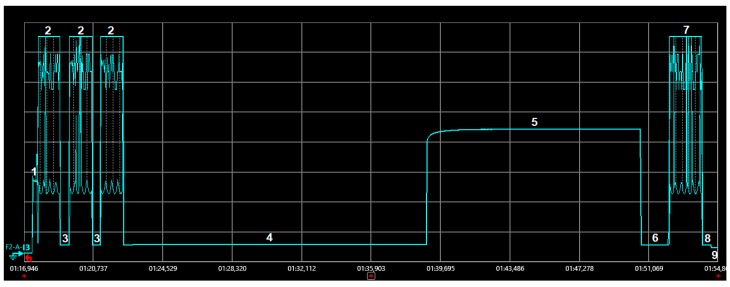
Current consumption profile of a MKRFOX1200 device during a bidirectional transaction initiated by the device. The uplink data message transmitted has a payload size of 1 byte.

This subsection models the average current consumption of a device that initiates bidirectional transactions periodically, *I_avg_bi_*. The model is derived from measurements on the corresponding current consumption profile of the same hardware platform, and with the same environment and methodology, used in the previous subsection. Figure 6 illustrates the current consumption of a device that initiates and successfully completes a bidirectional transaction.

In a bidirectional transaction, the number of states involved increases compared to that of a unidirectional transaction. Table 3 summarizes the states that correspond to a bidirectional transaction, as well as their related duration and current variables and values. The state duration and current values shown in Table 3 correspond to the average of 10 individual measurements in each case, with a maximum deviation from the average value below 6%.

The bidirectional transaction starts with the device transmitting the three replicas of its uplink message. Therefore, states 1–3 as described for unidirectional transactions (Table 2) are also present at the beginning of the bidirectional transaction. However, after the transmission of the third replica, the device waits (state 4) until it starts a reception interval (state 5). Once the device receives the downlink message sent by the base station, it waits for a shorter interval (state 6) until it sends the uplink confirmation. Shortly after, the device goes through a cool down phase (state 8), after which it returns to sleep mode (state 9).

In the absence of frame losses, the average current, *I_avg_bi_*, can be obtained by using Equations (8) and (9):

(8) $$I_{avg\_bi}=\frac{1}{T_{Period}}\sum_{j=1}^{N_{states\_bi}}n_{j}\cdot T_{j}\cdot I_{j}$$

(9) $$T_{sleep\_bi}=T_{Period}-T_{act\_bi}$$

where *T_j_* and *I_j_* represent the duration and current consumption of state *j* in Table 3, respectively, and *n_j_* indicates the number of times state *j* is present in the uplink data frame transmission procedure (note that *n*_2_ = 3, *n*_3_ = 2, and *n_j_*= 1 otherwise). Equations (8) and (9) are equivalent to (1) and (2), however considering now the states described in Table 3, using the number of states that corresponds to bidirectional transactions, *N_states_bi_* (equal to 9), and the time related with bidirectional transaction activities, *T_act_bi_*. The latter is computed as per the next equation:

(10) $$T_{act\_bi}=T_{wu}+3\cdot T_{tx}+2\cdot T_{wntx}+T_{wnrx}+T_{rx}+T_{wctrl}+T_{ctrl\_tx}+T_{cd}$$

Note that the base station has flexibility to determine the start of a downlink transmission. However, such transmission needs to fit the interval defined by the downlink window, with maximum duration denoted *T_DL_WIN_MAX_*. Assuming that all possible downlink start times are equally probable, *T_rx_* can be modeled as the expected value of a uniformly distributed random variable within the interval [*T_tx_DL_*, *T_DL_WIN_MAX_*]. Therefore, *T_rx_* is defined by the following equation:

(11) $$T_{rx}=\frac{T_{tx\_DL}+T_{DL\_WIN\_MAX}}{2}$$

Next, *I_avg_bi_* is determined in the presence of a non-zero FLR. Three different device current consumption profiles need to be considered, corresponding to three events that may occur, which are denoted *A*, *B* and *C*. Let *A* be the event where at least one of the three uplink frame transmissions is correctly received by the base station, and the downlink frame is also correctly received by the device. In this case, device current consumption is illustrated by Figure 6 and Table 3. Let *B* correspond to the event where the downlink frame is incorrectly received, and therefore the final uplink confirmation is not sent by the device. Finally, let *C* denote the event where none of the three uplink frame transmissions is correctly received, thus the base station does not send a downlink message, and therefore the device remains listening during the maximum downlink window duration. Based on the three events described, *I_avg_bi_* can be computed as shown next:

(12) $$I_{avg\_bi}=I_{A}\cdot p_{A}+I_{B}\cdot p_{B}+I_{C}\cdot p_{C}$$

where *I_A_*, *I_B_* and *I_C_* correspond to the average current consumption over a transaction period where events *A*, *B* and *C* occur, respectively, and *p_A_*, *p_B_*, and *p_C_* denote the corresponding respective probabilities. These current consumption and probability variables can be calculated as described next.

*I_A_* corresponds to the calculation of *I_avg_bi_* as determined by using Equations (8)–(11), since both the uplink and the downlink frame transmissions are successful in event *A*.

*I_B_* can be obtained similarly to (8), although the states related with sending the uplink confirmation, i.e., states 6 and 7 in Table 3, need to be excluded from the calculation as shown next:

(13) $$I_{B}=\frac{1}{T_{Period}}\left(\sum_{j=1}^{N_{states\_bi}}T_{j}\cdot I_{j}-\sum_{j=6}^{7}T_{j}\cdot I_{j}\right)$$

where state 9 in event *B*, i.e., the sleep interval in event *B*, denoted *T_sleep_bi_B_*, is calculated by using (14) and (15):

(14) $$T_{sleep\_bi\_B}=T_{Period}-T_{act\_bi\_B}$$

(15) $$T_{act\_bi\_B}=T_{wu}+3\cdot T_{tx}+2\cdot T_{wntx}+T_{wnrx}+T_{rx}+T_{cd}$$

Finally, *I_C_* can be determined on the basis of (13)–(15), but setting *T_rx_* to the maximum duration of the downlink receive window, denoted *T_DL_WIN_RX_MAX_*, since in event *C* the device stays in receive mode for that time. 

The probabilities *p_A_*, *p_B_*, and *p_C_*, can be calculated as follows:

(16) $$p_{A}=\left(1-FLR_{UL}{}^{3}\right)\cdot \left(1-FLR_{DL}\right)$$

(17) $$p_{B}=\left(1-FLR_{UL}{}^{3}\right)\cdot FLR_{DL}$$

(18) $$p_{C}=FLR_{UL}{}^{3}$$

where *FLR_DL_* denotes the FLR for downlink transmission. 

With regard to the rest of performance parameters considered in this paper, let *T_lifetime_bi_* and *EC_delivery_bi_* denote the device lifetime and the energy cost of data delivery, respectively, for a device that triggers bidirectional transactions. These performance parameters can be obtained on the basis of Equations (5) and (6), by replacing *I_avg_uni_* by *I_avg_bi_* in both equations, as shown next:

(19) $$T_{lifetime\_bi}=\frac{C_{battery}}{I_{avg\_bi}+I_{self\_dis}}$$

(20) $$EC_{delivery\_bi}=\frac{I_{avg\_bi}\cdot V\cdot T_{Period}}{E\left[l_{delivery}\right]}$$

Note that the expected amount of data delivered by the device, *E*[*l_delivery_*], is the same in either a unidirectional transaction or in a bidirectional transaction.

## 5. Evaluation

Based on the models presented in Section 4, this section evaluates Sigfox device current consumption and lifetime, as well as the energy cost of data delivery. This section is divided in five subsections. The first three focus on one of the aforementioned performance parameters, respectively. Section 5.4 analyzes the impact of critical parameters on the model. Finally, Section 5.5 studies the performance of Sigfox devices when using energy harvesting systems as power sources.

The evaluation considers a wide range of *T_Period_* values, including values (below 10 min) that are smaller than the minimum ones that stem from the maximum uplink and downlink message rate limitations intended to comply with spectrum access regulations. The purpose is illustrating the performance that can be achieved when such regulations are not in force (e.g., in some regions of the world).

### 5.1. Device Current Consumption

**Figure 7:**
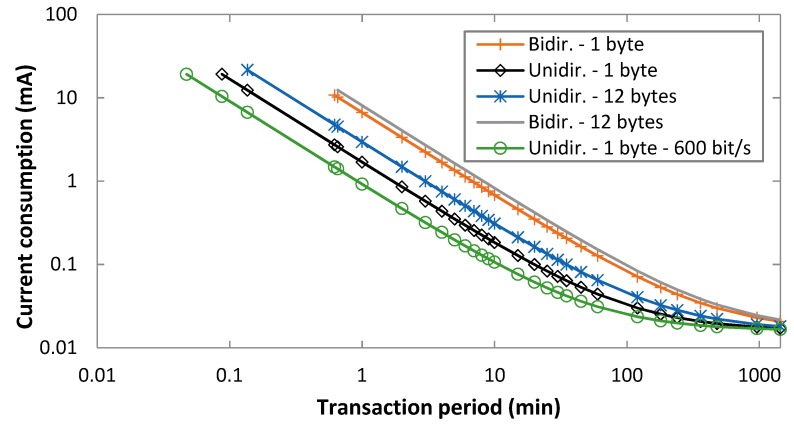
Average current consumption of the device, for unidirectional and bidirectional transactions, as a function of *T_Period_*, for FLR = 0, and for uplink payload sizes of 1 byte and 12 bytes.

This subsection evaluates the average Sigfox device current consumption, based on Equations (1)–(4) and (8)–(18). Figure 7 shows the average current consumption of the Sigfox device for both unidirectional and bidirectional transactions, as a function of *T_Period_*, for an uplink bit rate of 100 bit/s, FLR = 0, and uplink message payload sizes of 1 byte and 12 bytes. An uplink bit rate of 600 bit/s has also been evaluated; however, for the sake of illustration clarity, the corresponding curve is only shown for a 1-byte payload and unidirectional transactions in Figure 7. As expected, the current consumption decreases with the transaction period. When the latter is greater than 1000 min, the sleep interval becomes dominant, therefore differences among the considered options become reduced. Otherwise, unidirectional transactions yield significant current consumption savings, which increase as the transaction period decreases. For example, the average current consumption is 0.18 mA and 0.68 mA, for 1-byte payload unidirectional and bidirectional transactions, respectively, for *T_Period_* = 10 min.

Impact of the payload size on current consumption is relatively greater for unidirectional transactions, since bidirectional transactions comprise a downlink message and a final uplink confirmation that are independent of the data payload size in the data message sent. Finally, using a 600 bit/s uplink bit rate reduces significantly the current consumption for low to moderate transaction periods, especially for unidirectional transactions. The reason is an uplink frame transmission time decrease by a factor of 6, when compared to using 100 bit/s. For example, for *T_Period_* = 10 min, unidirectional transactions and a 1-byte payload, the average current consumption is 0.18 mA and 0.11 mA for uplink bit rates of 100 bit/s and 600 bit/s, respectively. The relative impact of the uplink bit rate decreases for bidirectional transactions (from 0.82 mA to 0.74 mA for a 1-byte payload, for uplink bit rates of 100 bit/s and 600 bit/s), and it decreases for both unidirectional and bidirectional transactions as *T_Period_* increases, where the sleep interval becomes dominant.

**Figure 8:**
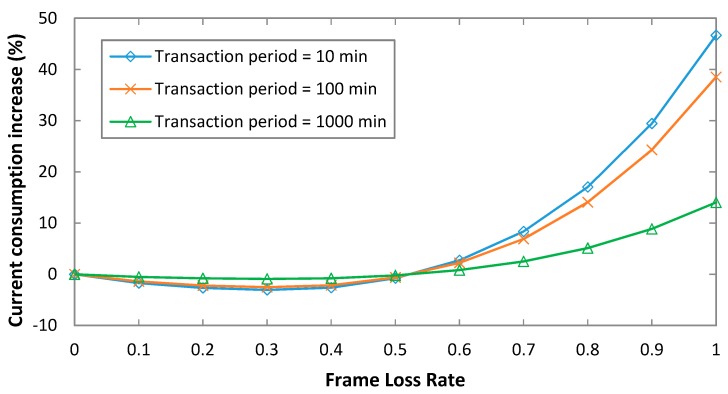
Impact of FLR on the average current consumption of the device, for bidirectional transactions, for an uplink bit rate of 100 bit/s, for an uplink payload size of 1 byte, and for different values of *T_Period_*.

We next evaluate the impact of a non-zero FLR on the current consumption of the device in bidirectional transactions, for an uplink payload size of 1 byte, and an uplink bit rate of 100 bit/s (Figure 8).

For simplicity, symmetric link performance is assumed, so that *FLR_UL_* = *FLR_DL_*. As shown in Figure 8, for relatively low FLR values, current consumption of the device slightly decreases (current consumption decrease is below 3%). This happens because in such region of FLR values, event *B* gains non-negligible influence. In this event, the downlink message is lost and the final uplink transmission is not performed, therefore reducing device current consumption (note that, while the uplink message is successfully delivered, the downlink one is not). For FLR values greater than 0.5, current consumption increases, since event *C* (i.e., none of the three uplink data frame transmissions is successful) becomes significant. In that event, the uplink transmission is not received by the base station, then there is no downlink message sent in response, and the device stays listening for the whole duration of the downlink receive window. However, in a real deployment, it is expected that FLR values should be reasonably low. Finally, note that the relative impact of FLR on current consumption decreases with the transaction period, since then the sleep interval becomes more dominant. 

### 5.2. Device Lifetime 

On the basis of Equations (5) and (19), and the results presented in the previous subsection, we next determine the device lifetime, for the same range of scenarios considered in the previous subsection. A battery with a capacity of 2400 mAh, and a self-discharge rate of 1%/year of its initial capacity is assumed [22].

**Figure 9:**
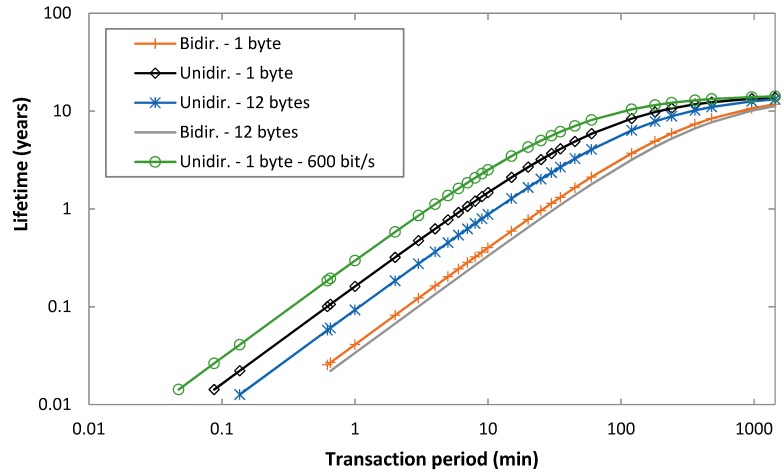
Device lifetime, for unidirectional and bidirectional transactions, as a function of *T_Period_*, and for uplink payload sizes of 1 byte and 12 bytes, for FLR = 0.

Figure 9 shows the lifetime of a Sigfox device under the assumed conditions, for both unidirectional and bidirectional transactions, as a function of *T_Period_*, for an uplink bit rate of 100 bit/s, for uplink message payload sizes of 1 byte and 12 bytes, and for FLR = 0. An uplink bit rate of 600 bit/s has also been evaluated; however, for the sake of illustration clarity, the corresponding curve is only shown for a 1-byte payload and unidirectional transactions. Overall, device lifetime behavior is inversely proportional to that of current consumption shown in the previous section. As expected, device lifetime increases with the transaction period, with an asymptotic device lifetime of 14.6 years. Differences between the considered options become negligible for a transaction period greater than 1000 min or more, since sleep interval then becomes dominant. Otherwise, differences among the considered options are significant, and they increase (in relative terms) as the transaction period decreases. For example, for a transaction period of 1000 min, and an uplink bit rate of 100 bit/s, device lifetime for unidirectional transactions is 13.4 years and 12.6 years, for 1-byte and 12-byte payloads, respectively. However, for a transaction period of 10 min and the same uplink bit rate of 100 bit/s, device lifetime for unidirectional transactions is 1.47 years and 0.87 years, respectively. As it can be seen, impact of the uplink message payload size on device lifetime is significant, especially for unidirectional transactions, and for low to moderate transaction periods (of up to ~100 min). On the other hand, use of bidirectional transactions significantly reduces device lifetime compared to unidirectional transactions, especially for low to moderate transaction periods. For example, for a 1-byte payload, an uplink bit rate of 100 bit/s and a transaction period of 10 min, device lifetime is 1.47 years and 0.40 years for unidirectional transactions and for bidirectional transactions, respectively. Finally, using an uplink bit rate of 600 bit/s increases device lifetime for low to moderate transaction periods. For example, for *T_Period_* = 10 min, unidirectional transactions and a 1-byte payload, the device lifetime is 1.47 years and 2.51 years for uplink bit rates of 100 bit/s and 600 bit/s, respectively. Similarly to the behavior of the average current consumption as the transaction period increases, the relative impact of the uplink bit rate decreases for bidirectional transactions (e.g., from 0.40 years to 0.43 years for a 1-byte payload, for uplink bit rates of 100 bit/s and 600 bit/s and *T_Period_* = 10 min), and it decreases for both unidirectional and bidirectional transactions with the transaction period.

With regard to non-zero FLR scenarios, the relative impact on device lifetime can be determined on the basis of the results shown in Figure 8. Since device lifetime is inversely proportional to current consumption, the relative impact of FLR on device lifetime is the inverse of the one shown in Figure 8. Device lifetime slightly increases (by a factor below 3%) for low FLR values (at the expense of downlink message loss) and it decreases for very low quality links (FLR > 0.5).

### 5.3. Energy Cost of Data Delivery 

**Figure 10:**
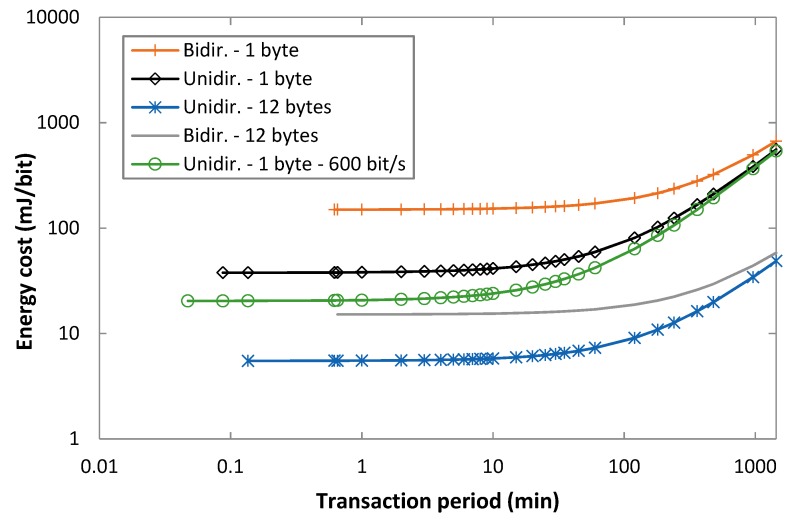
Energy cost of data delivery, for unidirectional and bidirectional transactions, as a function of *T_Period_*, and for uplink payload sizes of 1 byte and 12 bytes, for FLR = 0.

This subsection evaluates the energy cost of data delivery for both unidirectional and bidirectional transaction types, on the basis of Equations (6) and (20). Figure 10 depicts *EC_delivery_uni_* and *EC_delivery_bi_* as a function of the transaction period, for unidirectional and bidirectional transactions, for 1-byte and 12-byte payload sizes, and for uplink bit rates of 100 bit/s and 600 bit/s. For the sake of illustration clarity, from the 600 bit/s uplink bit rate cases, only the curve corresponding to a 1-byte payload and unidirectional transactions is shown. For all considered options, the energy cost of data delivery increases with the transaction period. The reason is that one data payload is delivered per transaction period, and the energy consumption over a transaction period increases with the latter (due to the current consumption during a sleep interval that also increases). As the transaction period increases, the difference between using unidirectional and bidirectional transactions becomes less relevant, since the sleep interval becomes dominant. 

A remarkable result is that, for an uplink bit rate of 100 bit/s, the energy cost of delivering a 12-byte payload with a bidirectional transaction is from 2 to 10 times lower than that of delivering a 1-byte payload with a unidirectional transaction. As expected, the energy cost of data delivery is inversely proportional to the delivered payload size.

Finally, the energy cost of data delivery decreases for an uplink bit rate of 600 bit/s, compared to that obtained for an uplink bit rate of 100 bit/s, since transmission time of the uplink messages decreases as well. The quantitative impact of the uplink bit rate is more relevant for unidirectional transactions than for bidirectional ones. For example, for a 1-byte payload, *T_Period_* = 10 min, and an uplink bit rate of 100 bit/s, the energy cost of data delivery in unidirectional transactions is 1.59 times greater than that obtained with an uplink rate of 600 bit/s. However, for the same settings but using bidirectional transactions, the relative difference decreases to a factor of 1.06. As the transaction period increases, impact of the bit rate tends to decrease since the active states become less relevant.

**Figure 11:**
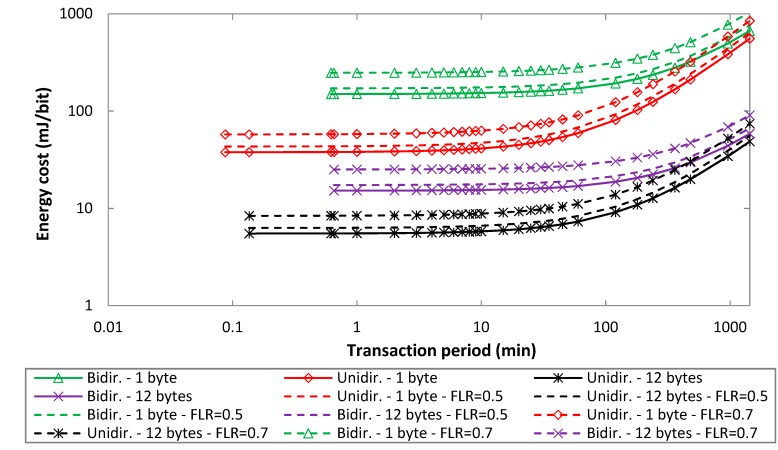
Energy cost of data delivery, for unidirectional and bidirectional transactions, as a function of *T_Period_*, for uplink payload sizes of 1 byte and 12 bytes, for an uplink bit rate of 100 bit/s, and for different FLR values.

In order to better assess the impact of loss rate on both *EC_delivery_uni_* and *EC_delivery_bi_*, Figure 11 shows both performance parameters, for unidirectional and bidirectional transactions, for an uplink bit rate of 100 bit/s, and for different FLR values. As the FLR grows, the energy cost of data delivery increases. For unidirectional transactions, while device current consumption is independent of the frame loss rate, the energy cost of data delivery increases with FLR. For bidirectional transactions, as explained in Section 5.1, FLR leads to a slight decrease in device current consumption for FLR up to ~0.5, which is compensated by the corresponding lower delivery rate to keep a roughly constant energy cost of data delivery as a function of FLR. For greater FLR values, energy cost of data delivery increases; since current consumption increases, while delivery rate decreases, the energy cost of data delivery for bidirectional transactions exhibits a greater increase with FLR than for unidirectional transactions. For example, for FLR = 0.7, *EC_delivery_uni_* and *EC_delivery_bi_* increase by up to 52% and 64%, respectively, when compared to FLR = 0.

### 5.4. Impact of Critical Parameters on the Model 

This subsection studies the impact of critical parameters, such as the sleep current, the Sigfox device transmit power, and the uplink frame size, on the model presented in this paper.

**Figure 12:**
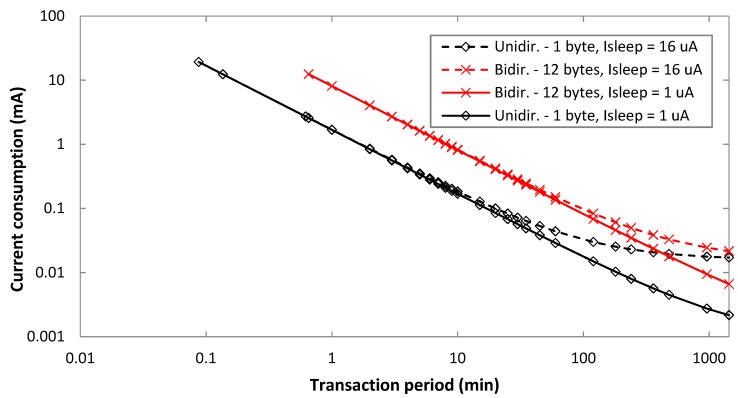
Average current consumption of the device, for different transaction types and uplink payload sizes, as a function of *I_sleep_*, for FLR = 0.

A parameter with a crucial impact on energy consumption metrics is the sleep current, *I_sleep_*. As discussed in Section 4.1, the sleep current in the device used for this work is 16 µA, whereas transceiver datasheets may indicate a sleep current in the order of 1 µA. Figure 12 depicts the average current consumption for *I_sleep_* settings such as 16 µA and 1 µA, for different transaction types, and FLR = 0. An uplink bit rate of 100 bit/s is assumed. As it can be seen, impact of *I_sleep_* increases with the transaction period, since *I_sleep_* is the asymptotic current consumption value (note that *I_self_dis_* needs to be considered as well when a battery is used, with a relative impact that increases as the transaction period decreases). For bidirectional transactions, and a 12-byte payload, the *I_sleep_* settings considered lead to significant current consumption differences for a *T_Period_* of around 100 min or greater. For unidirectional transactions, and a 1-byte payload, current consumption differences arise for lower *T_Period_* values (e.g., 10 min), due to the lower overall current consumption in unidirectional transactions.

Another parameter that is relevant in terms of energy consumption is the Sigfox device transmit power. The device used in our evaluation only supports one transmit power value (i.e., 14.5 dBm). Such a value matches the maximum allowed device transmit power according to EU regulations, and for this reason it is commonly used as the default or the only transmit power setting in other Sigfox hardware platforms [9,10,11,13]. However, from the point of view of the model, the transmit power used has an influence on the performance parameters considered in this paper, since *I_tx_* depends on the transmit power setting.

**Figure 13:**
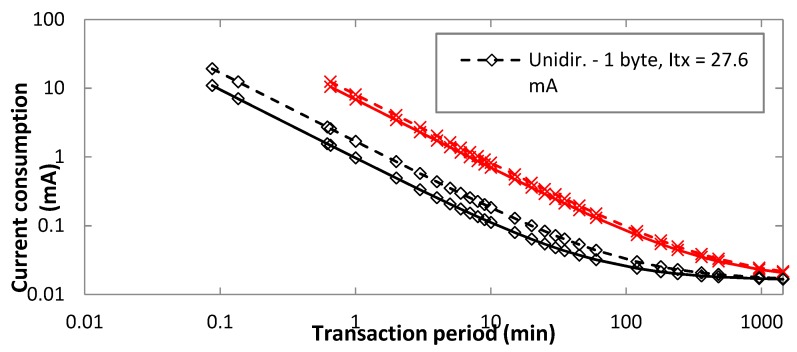
Average current consumption of the device, for different transaction types, as a function of *I_tx_*, for FLR = 0, and for different uplink payload size of 1 byte.

Figure 13 illustrates the impact on the average current consumption of considering different *I_tx_* values, such as the one obtained with our measurements, i.e., 27.6 mA, and 15 mA, different transaction types and uplink payload sizes. An uplink bit rate of 100 bit/s is assumed. As shown in Figure 13, the transmit power, and thus *I_tx_*, is relevant for low transaction periods. The impact of *I_tx_* is greater for unidirectional transactions, since in bidirectional transactions the Sigfox device consumes a significant amount of current during the receive window. 

Finally, another relevant parameter is the uplink frame size. Note that this parameter grows linearly with the uplink frame payload size and, as already illustrated in Section 5.1, Section 5.2 and Section 5.3, it has a relevant impact on device current consumption, device lifetime and energy cost of data delivery. However, another important aspect to consider regarding the uplink frame size is its relationship with the FLR. A frame may be lost due to bit errors (e.g., due to poor received signal, challenging signal propagation conditions, interference, etc.), and it may also be lost due to collisions [7]. Increasing the uplink frame size increases the FLR for both reasons.

As shown in Section 5.1 and Section 5.3, the FLR affects current consumption for bidirectional transactions (mostly increasing current consumption for *FLR* > 0.5), and a non-zero FLR increases the energy cost of data delivery.

### 5.5. Use of Energy Harvesting 

While using a battery as the energy source for a sensor device is a common approach, energy harvesting is also a relevant alternative. This section evaluates the minimum feasible transaction period for a Sigfox device powered by an energy harvesting source. The results are obtained based on Equations (1)–(4) and (8)–(18), considering 1-byte and 12-byte payloads, unidirectional and bidirectional transactions, and two energy harvesting system models: a Panasonic AT-7665A film outdoor solar panel, and a Panasonic AM-1815CA glass indoor solar panel [23,24]. The former typically provides a current of 38.6 mA and a voltage of 3 V, whereas the latter typically gives 47 µA and 3 V.

Table 4 shows, for each considered transaction type, for an uplink bit rate of 100 bit/s, and for the two considered energy harvesting sources, the energy required to carry out the transaction, and the minimum feasible transaction period. The device is assumed to be in sleep state between transactions. The outdoor solar panel allows using all *T_Period_* values considered in this paper, i.e., it does not limit by itself the minimum feasible *T_Period_*. However, the indoor panel supplies a much lower current (three orders of magnitude below the outdoor panel one). This requires accumulating the energy harvested by the indoor panel over relatively large periods of time before allowing completing a transaction, up to ~1.5 h or ~4 h for unidirectional and bidirectional transactions, respectively.

## 6. Conclusions

This paper has presented analytical models that allow to evaluate the current consumption and the lifetime of a battery-enabled Sigfox device, as well as the energy cost of data delivery. The model, which has been derived based on measurements performed on real hardware, captures the impact of using uplink and downlink communication, the frame payload size, the uplink bit rate, and frame losses on the aforementioned energy performance parameters.

The average current consumption decreases with the transaction period, with an asymptotic value equal to the sleep state current consumption. Differences among the considered options decrease as well with the transaction period. For low transaction periods, bidirectional communication increases current consumption by a factor up to ~4. Current consumption is only affected by frame losses for bidirectional transactions. In those, since an uplink frame is sent thrice, the impact of frame losses becomes only relevant for very high FLR values, where the device awaits a downlink frame for the maximum receive window duration. 

Assuming a 2400 mAh battery, and the device model used in the study, the theoretical asymptotic device lifetime is 14.6 years. Similarly to the observations made for current consumption, differences between using bidirectional or unidirectional transactions are significant for low transaction periods. For an uplink bit rate of 100 bit/s, a 1-byte payload and a transaction period of 10 minutes, device lifetime is 1.49 years and 0.40 years for unidirectional transactions and for bidirectional transactions, respectively. Using an uplink bit rate of 600 bit/s (in regions where that is possible) increases device lifetime for low to moderate transaction periods. For a transaction period of 10 min, unidirectional transactions and a 1-byte payload, the device lifetime is 1.49 years and 2.57 years for uplink bit rates of 100 bit/s and 600 bit/s, respectively.

The uplink frame payload size has a greater impact on the energy cost of data delivery than on the rest of performance parameters considered. Increasing the uplink frame payload size amortizes the energy consumed per delivered bit by a factor similar to the payload size increase. Using an uplink bit rate of 600 bit/s leads to remarkable energy cost savings (up to a factor of 1.84). Frame losses impact on the energy cost for rather high FLR values (of 0.5 or greater) for both unidirectional and bidirectional transactions, to a greater extent for the latter.

The paper also evaluates the sensitivity of the current consumption model to *I_sleep_* and *I_tx_*, two relevant parameters. Finally, energy harvesting sources have been considered as well, illustrating that powerful ones allow the operation of the Sigfox device for the whole range of transaction types and periods considered in this paper, whereas more limited energy harvesting sources constrain the range of feasible transaction periods.

## Figures and Tables

**Table 1:** Current consumption of Sigfox devices considered in the literature. For each referenced paper, the device and the current consumption states (names and values) are shown, indicating their source. The work by Martínez et al. is not included because current consumption states are not identified and their related values are not provided in that work [15].

Reference	Device Name	Current Consumption: Uplink (left) and Downlink (right)				Source of Values
		State Name	Value (mA)	State Name	Value (mA)	
[9]	Not specified	Transmission	49	Reception	13	Not specified
		Sleep	1.44 × 10^−3^			
[10]	ONSEMI AX-Sigfox	Tx (0 dBm)	19	-	-	Datasheet [16]
		Tx (14 dBm)	49			
		Standby	0.5			
		Sleep	1.3 × 10^−3^			
[11]	Telit LE51-868/DIP	Cmd	8.4	Cmd	8.4	Empirical
		Tx	54	Tx	54	
		Delay Tx	12	Delay Tx	12	
		Standby	<0.1 (a)	Standby	<0.1	
				RxW	34	
				Delay Rx	8.4	
				Rx	34	
[12]	TD1207R/08R	Transmission	50	Reception	13	Datasheet [17]
[13]	ONSEMI AXSFEU	Transmission	49	Reception	10	Datasheet [18]
[14]	FiPy	Wake up 1	80	-	-	Empirical
		Wake up 2	160			
		Idle	280			
		Tx	0.32/0.42			
		Deep sleep	25 × 10^−3^			

**Table 2:** States, variables and measurement results for Sigfox unidirectional transactions. For a bit rate of 100 bit/s, *T_tx_* can take values between 1200 ms (1-byte payload) and 2080 ms (12-byte payload). *T_sleep_uni_* ranges in this study from 0 up to ~*T_Period_* (*T_sleep_uni_* tends to *T_Period_* for very high *T_Period_* values).

State Number	Description	Duration		Current Consumption	
		Variable	Value (ms)	Variable	Value (mA)
1	Wake up	*T_wu_*	287	*I_wu_*	10.4
2	Transmission	*T_tx_*	[1200,2080] (4)	*I_tx_*	27.2
3	Wait next transmission	*T_wntx_*	486	*I_wntx_*	1.2
4	Cool down	*T_cd_*	510	*I_cd_*	1.2
5	Sleep	*T_sleep_uni_*	[0,*T_Period_*) (2)	*I_sleep_*	16 × 10^−3^

**Table 3:** States, variables and their values for Sigfox bidirectional transaction. For a bit rate of 100 bit/s, *T_tx_* can take values between 1200 ms (1-byte payload) and 2080 ms (12-byte payload). The reception duration ranges from 387 ms up to *T_DL_WIN_MAX_* = 25 s (in US and EU regions), with an average of *T_rx_*= 12.69 s. *T_sleep_bi_* ranges in this study from 0 up to ~*T_Period_* (*T_sleep_bi_* tends to *T_Period_* for very high *T_Period_* values).

State Number	Description	Duration		Current Consumption	
		Variable	Value (ms)	Variable	Value (mA)
1	Wake up	*T_wu_*	305	*I_wu_*	10.7
2	Transmission	*T_tx_*	[1200,2080] (4)	*I_tx_*	27.6
3	Wait next transmission	*T_wntx_*	493	*I_wntx_*	1.2
4	Wait next reception	*T_wnrx_*	16493	*I_wnrx_*	1.3
5	Reception	*T_rx_*	12690 (11)	*I_rx_*	18.5
6	Wait confirm. transmission	*T_wctrl_*	1430	*I_wctrl_*	1.2
7	Confirmation transmission	*T_ctrl_tx_*	1850	*I_ctrl_tx_*	27.0
8	Cool down	*T_cd_*	495	*I_cd_*	1.2
9	Sleep	*T_sleep_bi_*	[0,*T_Period_*) (9)	*I_sleep_*	16 × 10^−3^

**Table 4:** Energy required and minimum feasible *T_Period_* for different types of transactions, for the two energy harvesting sources considered, and for an uplink bit rate of 100 bit/s.

Transaction	Energy Required (mJ)	Outdoor Panel: Minimum *T_Period_* (min)	Indoor Panel: Minimum *T_Period_* (min)
1-byte, unidirectional	2.61	0.09	54.1
12-byte, unidirectional	4.56	0.14	94.7
1-byte, bidirectional	10.3	0.62	214.6
12-byte, bidirectional	12.6	0.65	261.2
